# Hippocampal DNA Methylation in a Mouse Model of Fetal Alcohol Spectrum Disorder That Includes Maternal Separation Stress Only Partially Explains Changes in Gene Expression

**DOI:** 10.3389/fgene.2020.00070

**Published:** 2020-02-27

**Authors:** Bonnie L.J. Alberry, Shiva M. Singh

**Affiliations:** Department of Biology, Western University, London, ON, Canada

**Keywords:** fetal alcohol spectrum disorder, prenatal alcohol, hippocampus, maternal separation, DNA methylation, early life stress

## Abstract

Fetal alcohol spectrum disorder (FASD) is characterized by developmental and behavioral deficits caused by maternal drinking during pregnancy. Children born with FASD often face additional stresses, including maternal separation, that add yet additional deficits. The mechanism associated with this interaction is not known. We have used a mouse model for prenatal ethanol exposure and maternal separation to demonstrate that the combination of the two treatments results in more than additive deficits. Furthermore, the behavioral deficits are associated with changes in hippocampal gene expression that persist into adulthood. What initiates and maintains these changes remains to be established and forms the focus of this report. Specifically, MeDIP-Seq was used to assess if changes in promoter DNA methylation are affected by exposure to prenatal ethanol and maternal separation including its relationship to gene expression. The novel results show that different sets of genes implicated by promoter DNA methylation are affected by both treatments independently, and a relatively unique set of genes are affected by the combination of the two treatments. Prenatal ethanol exposure leads to altered promoter DNA methylation at genes important for transcriptional regulation. Maternal separation leads to changes at genes important for histone methylation and immune response, and the combination of two treatments results in DNA methylation changes at genes important for neuronal migration and immune response. Our dual results from the same hippocampal samples suggest there is minimal complementarity between changes in promoter DNA methylation and gene expression, although genes involved tend to be critical for brain development and function. While remaining to be validated, such results argue that mechanisms beyond promoter DNA methylation must be involved in lasting gene expression alterations leading to behavioral deficits implicated in FASD. They may facilitate early and reliable diagnosis, as well as novel strategies for the amelioration of FASD-related deficits.

## Introduction

Fetal alcohol spectrum disorder (FASD) is a serious neurodevelopmental disorder that begins in utero, manifests during childhood, and lasts a lifetime. It is caused by prenatal ethanol exposure primarily by maternal gestational alcohol consumption and consists of a collection of conditions including developmental delays, restrictions, abnormalities, as well as intellectual and behavioral impairments (Chudley et al., 2005). The incidence of FASD varies across different populations, with the estimated prevalence in 7- to 9-year-old Canadians is between 2% and 3% (Popova et al., 2018), and between 1% and 5% in the United States (May et al., 2018). This incidence is expected to increase given a recent Canadian report that alcohol-related emergencies between 2003 and 2016 rose 4.4 times more than overall emergency visits (Myran et al., 2019). Specifically, this increase is greater for women (86.5%) than men (53.2%). Among women, 15- to 24-year-olds have the highest rate. Of the medical harms identified, suspected fetal damage including fetal alcohol syndrome rose 2133.3% from 2003 to 2016. As it may extend to other populations, this trend is concerning for more than just Ontario and does not bode well for a focus on FASD prevention *via* alcohol avoidance. The next best strategy is to offer interventions following early diagnosis, which remains problematic given the heterogeneity of manifestation and lack of reliable biomarker. As such, FASD continues to represent a major health and social burden in alcohol-friendly societies.

Given that prenatal alcohol exposure is the cause of FASD, and neurodevelopment is long-lasting, spanning decades, research must also focus on postnatal factors that may determine the manifestation of the disorder. Furthermore, this understanding may offer novel strategies for the remediation of FASD deficits and severity. Molecular brain research in humans is restrictive and must rely on animal models. Our lab has developed a mouse model of FASD using C57BL/6J (B6) mice (Kleiber et al., 2011), establishing that FASD is like an iceberg. Ethanol-exposed pups develop learning deficits, anxiety-like behaviors, and altered activity patterns (Allan et al., 2003; Kaminen-Ahola et al., 2010; Kleiber et al., 2011; Marjonen et al., 2015). What we see as deficit(s) is just the tip—most underlying defects are not visible and are reflected in gene expression alterations (Kleiber et al., 2012).

Mammalian neurodevelopment involves the orchestration of cellular processes that are sensitive to prenatal and postnatal environmental stresses over time (Tau and Peterson, 2010). One common postnatal stress that children born with FASD face is maternal separation. Children entering childcare systems, such as foster care or orphanages, represent a particularly vulnerable population, with FASD considerably overrepresented in such individuals (Lange et al., 2013). In Canada, the prevalence of FASD is 5 to 67 times higher than a global estimate (Lange et al., 2017). In addition to the initial gestational alcohol exposure, exposure to early life stress increases the risk of behavioral deficits, including adult psychiatric disorders (Kisely et al., 2018). Early life stress has a negative effect on learning and memory processes governed by the hippocampus in rodents (Rice et al., 2008; Oomen et al., 2010; Pillai et al., 2018). Little is known about how early life stress may affect children born with FASD (Price et al., 2017), although following gestational alcohol exposure and abuse or neglect during early development, children are more likely to have behavioral deficits, including impaired learning and memory (Coggins et al., 2007; Henry et al., 2007; Koponen et al., 2009; Koponen et al., 2013).

Since children with FASD often face additional early life stress, we have extended the model to explore how postnatal maternal separation may compound developmental and behavioral deficits following prenatal ethanol exposure (Alberry and Singh, 2016). The behaviors affected by prenatal alcohol exposure and maternal separation include anxiety-like behavior as well as learning and memory. While many brain regions may be relevant, the hippocampus forms the focus of this study for its essential role in spatial learning (Jarrard, 1993). To this end, we have also reported changes in hippocampal gene expression that persist into adulthood following prenatal ethanol exposure combined with postnatal maternal separation in B6 mice (Alberry et al., 2019). Given that the initiation and maintenance of aberrant gene expression in response to prenatal ethanol exposure includes epigenetic mechanisms, primarily DNA methylation, noncoding RNA, and histone modifications (Chen et al., 2013; Laufer et al., 2013; Chater-Diehl et al., 2016), these processes are expected to be critical in other models of FASD. In fact, FASD-related human research in epigenetic mechanisms has begun to focus on DNA methylation (Laufer et al., 2015; Portales-Casamar et al., 2016). While there are many epigenetic mechanisms that may alter transcription, here we focus on promoter DNA methylation that may repress transcription *via* blocked transcription factor binding to explore the canonical relationship between gene expression and promoter DNA methylation (Jaenisch and Bird, 2003).

This research is novel in that it combines the effect of prenatal ethanol exposure and maternal separation on the FASD iceberg using B6 mice at three levels—behavior, hippocampal gene expression and hippocampal DNA methylation on the same samples. Although the behavioral results are compatible with molecular changes, changes in gene expression are rarely related to changes in promoter-specific methylation. It may represent a feature of the experimental design or reflect a general feature of the development of FASD. Although one is tempted to suggest that epigenetic changes other than DNA methylation must represent important underlying mechanisms involved in the development of FASD, the issue deserves further experimentation and comprehensive evaluation. Such clarifications will be needed in the use of these results, including the development of effective measures of remediation.

## Materials and Methods

### Animals

C57BL/6J mice originally obtained from Jackson Laboratories (Bar Harbor, ME) were bred and housed in same-sex colonies of up to four individuals with unrestricted food and water access at the Animal Care Facility at Western University (London, Ontario, Canada). Temperature and humidity were maintained at 21°C–24°C and 40%–60%, respectively, with lighting on a 14:10-h light-dark cycle. All housing, bedding, and nest material were consistent between cages. All treatment protocols complied with ethical standards established by the Canadian Council on Animal Care and were approved by the Animal Use Subcommittee at Western University (London, Ontario, Canada).

### Continuous Preference Drinking Model

Females (10 weeks old) were housed individually and randomly assigned to ethanol or control groups. Both groups had free access to water, with the ethanol group additionally provided free access to 10% ethanol in water solution (Kleiber et al., 2011; Alberry and Singh, 2016). Ethanol was introduced with increasing concentrations of 2%, 5%, and 10% after 48 h with the previous concentration. After 10 days of continuous 10% ethanol availability, females were mated with males (15 weeks old) with only water available. Males were removed after a 24-h period and 10% ethanol was returned, which represented gestational day 0. Until postnatal day 10, 10% ethanol was freely available, with only water available for the remainder of this study. Blood alcohol levels were not taken to minimize maternal stress, voluntary consumption of 10% ethanol at 14 g ethanol per kg body weight per day produces peak blood alcohol levels of 120 mg dl^-1^ (Allan et al., 2003). This study represents a modest amount of ethanol exposure, with experimental mice consuming 8 g ethanol per kg body weight per day on average, sufficient to produce behavioral deficits in their offspring, including hyperactivity in a novel environment, hypoactivity in a home cage environment, and learning deficits (Alberry and Singh, 2016).

### Early Life Stress *Via* Maternal Separation and Isolation

Postnatal maternal separation and isolation were used to induce early life stress, as described previously (Savignac et al., 2011; Benner et al., 2014; Alberry and Singh, 2016). On postnatal day 2, half of each litter was randomly selected for stress, tail coloring with permanent marker was used to distinguish pups. From postnatal days 2 to 14, stress group pups were removed and isolated for 3 hours per day during the light phase in 8 oz. Dixie cups with bedding and nest material. Pups not selected for maternal separation remained with the dam and other littermates during this time. On postnatal day 21, pups were weaned and housed with same-sex littermates with 2–4 individuals per cage. Following treatments, mice belonged to one of four groups—control, ethanol, stress, or ethanol + stress.

### Hippocampal Dissection and DNA Isolation

Male mice were sacrificed *via* carbon dioxide asphyxiation and cervical dislocation on postnatal day 70. The hippocampus was dissected from the whole brain as described previously (Spijker, 2011). With samples stored in phosphate-buffered saline (PBS), snap-frozen in liquid nitrogen, and kept at −80°C. Samples were ground by pestle over liquid nitrogen to create a powder. Stages of buffer RLT (Qiagen, Valencia, CA) were added and mixed by pipetting. Sampled were centrifuged after a 10-min incubation period. The supernatant was loaded onto AllPrep DNA spin columns and the AllPrep DNA/RNA Mini Kit Protocol (Qiagen, Valencia, CA) was followed to isolate DNA and RNA from the same sample. DNA quantification was determined by NanoDrop 2000c Spectrophotometer (Thermo Fisher Scientific, Wilmington, DE).

### MeDIP-Seq

Three DNA samples were randomly selected from each treatment group (12 total DNA samples from individual mice processed separately) and sent on dry ice to Arraystar Inc. (Rockville, MD), with one sample per group as an input sample for sequence control. DNA was quantified by NanoDrop 1000 (Thermo Fisher Scientific, Wilmington, DE), then fragmented to a range of 200–1,500 bp using a Diagenode Bioruptor, end-repaired, and 3' adenylated for ligation of genomic adapters. Fragments were immunoprecipitated by anti-5-methylcytosine antibody, then PCR amplified. AMPure XP beads were used to select fragments from 300–800 bp, then quantified by Agilent 2100 Bioanalyzer (Agilent Technologies, Santa Clara, CA). Following denaturation with 0.1 M NaOH, single-stranded DNA molecules were captured and amplified *in situ* on an Illumina flow cell. Libraries were sequenced on the Illumina HiSeq 4000 platform using the HiSeq 3000/4000 SBS Kit (300 cycles) protocol, then Off-Line Basecaller (OLB V1.8) was used for base calling. Reads were aligned to the mouse genome (UCSC MM10) using HISAT2 (V2.1.0) (Kim et al., 2015) after passing the Solexa CHASTITY quality filter and resulting BED files were used for differential methylation analysis. Sequences have been made available at GEO accession number GSE137984.

### Differential Methylation Analysis

Aligned reads from HISAT2 were used for peak calling using Model-based analysis for ChIP-Seq (MACS) (version 1.4.2) (Zhang et al., 2008), where enriched regions (peaks) for each sample were identified by comparison to input background samples for each group using a dynamic Poisson distribution (*q* < 10^-5^). MeDIP enriched regions were annotated to the nearest gene using UCSC RefSeq, with differentially methylated regions (DMRs) in the promoter of known genes for Ethanol vs. Control, Stress vs. Control, as well as Ethanol + stress vs. Control identified by diffReps (cut-off: log_2_FC = 1.0, *p* = 10^-4^) (Shen et al., 2013). Promoter regions are defined here as 2,000 bp upstream and downstream from the transcription start site of a gene. Generalized hypergeometric tests for enrichment of gene ontology (GO) terms and Kyoto encyclopedia of genes and genomes (KEGG) pathways were used for genes represented by promoter DMRs for each treatment group using goana and kegga functions in the limma software package (Ritchie et al., 2015) in R, filtered by significance (*p* < 0.05).

### Comparison to Transcriptome Analysis

Genes represented by promoter DMRs for each treatment group were compared to previously reported transcript-level expression analysis from an RNA-Seq experiment using RNA isolated from the same samples (Alberry et al., 2019). Briefly, RNA-Seq was carried out by The Centre for Applied Genomics (The Hospital for Sick Children, Toronto, Ontario, Canada). Paired-end reads were pseudo-aligned to version 38 of the Ensembl mouse transcriptome *via* kallisto (Bray et al., 2016) and differential expression analysis was determined using sleuth (Pimentel et al., 2017; Yi et al., 2018). Gene lists used here were filtered by significance (*p* < 0.01), resulting in a list of 164 unique transcripts altered following ethanol exposure, 116 for stress, and 217 for the combination of ethanol + stress. Sequence data for the RNA-Seq experiment is available at the Gene Expression Omnibus (GEO), accession number GSE133369.

## Results

Hippocampal DNA samples from adult males (postnatal day 70) for each of four groups of mice representing a control group with no experimental interventions, an ethanol group that faced prenatal ethanol exposure, a stress group that faced postnatal maternal separation stress, and an ethanol + stress group subject to both treatments (see methods for specific details). DNA methylation profiles were assessed *via* MeDIP-Seq to determine how treatment groups differ from controls.

### DNA Methylation Changes Following Different Treatments

**Figure 1:**
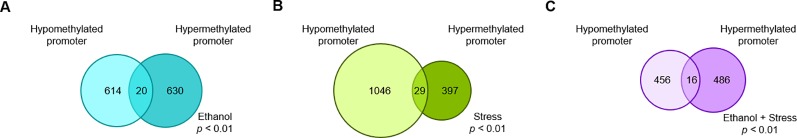
The number of genes with significant (*p* < 0.01) promoter DNA methylation changes following **(A)** prenatal ethanol treatment, **(B)** early life stress treatment, or **(C)** the combination of prenatal ethanol treatment and early life stress as compared to controls.

DMRs (DMRs) were determined for each experimental treatment group compared to controls, then associated with annotated genes *via* proximity to transcription start sites. Promoter regions are defined here as 2 kb upstream or downstream from the transcription start site of a gene, with genes with promoter DMRs considered for this analysis. In this way, several DMRs considered spatially distinct can be present in the promoter region of a single or multiple gene(s). A gene implicated by more than one DMR is considered separately when the DMRs are in opposition, but only once if the DMRs are in the same direction. Using promoter DMRs, 1,264 genes are implicated by prenatal ethanol treatment (**Figure 1A**, details in **Supplementary Table 1**). Following postnatal maternal separation stress, 1,472 genes are represented by promoter DMRs (**Figure 1B**, details in **Supplementary Table 2**). When mice faced both ethanol + stress, 958 genes are implicated by promoter DMRs (**Figure 1C**, details in **Supplementary Table 3**). Interestingly, while the ethanol group has a similar number of genes implicated by hypo- and hypermethylated promoter DMRs, the stress group genes are represented by 73% hypomethylated promoter DMRs. Genes implicated by promoter DMRs in the ethanol + stress group are also more evenly distributed between hypo- and hypermethylated. While a single DMR is either hypo- or hypermethylated, a single gene may be represented by multiple DMRs with different states. There are between 16 and 29 genes in each comparison that are implicated by both hypo- and hypermethylation of promoter DMRs.

Hypo- and hypermethylated DMR-associated genes for each treatment group were analyzed separately for GO and KEGG pathway enrichment. In the ethanol group, genes related to voltage-gated calcium channel activity tended to be hypomethylated at their promoter regions (*p* < 0.007) (**Table 1**). In addition, brain-related terms including NMDA receptor activity (*p* = 0.001), positive regulation of behavior (*p* = 0.002), circadian rhythm (*p* = 0.003), and apical dendrite (*p* = 0.03) were all biological processes overrepresented by genes with hypomethylated promoters following ethanol exposure. Interestingly, components responsible for transcriptional regulation are also implicated by genes with hypomethylated promoters, including PRC1 complex (*p* = 4.04 x 10^-4^), transcription factor TFIID complex (*p* = 0.03), and RNA polymerase II transcription factor complex (*p* = 0.046). Genes related to phosphoinositide kinase (*p* < 0.02) and cytoskeleton regulation (*p* = 0.005) were hypermethylated at their promoter regions following prenatal ethanol exposure. In the stress group, genes important for histone modifications (*p* < 0.01) and neuronal organization (*p* = 0.007) were overrepresented by genes with hypomethylated promoters (**Table 2**). Conversely, genes with hypermethylated promoters following stress are important for cytokine interactions (*p* < 0.006). When mice faced both ethanol + stress, genes important for neuron migration (*p* < 0.005) were hypomethylated at their promoters (**Table 3**). As with stress alone, the combination of ethanol + stress results in promoter hypermethylation for genes responsible for cytokine interactions (*p* < 0.01).

**Table 1:** Top five most significantly (*p* < 0.05) overrepresented gene ontology (GO) terms and Kyoto encyclopedia of genes and genomes (KEGG) pathways represented by either hypo- or hypermethylated differentially methylated region (DMR)-associated genes implicated by Ethanol treatment compared to control (*p* < 0.01).

Hypomethylated	p-value	Hypermethylated	p-value
Term		Term	
**Molecular function**			
hydrolase activity, hydrolyzing O-glycosyl compounds	0.001	phosphatidylinositol bisphosphate kinase activity	0.001
voltage-gated cation channel activity	0.005	phosphatidylinositol-4,5-bisphosphate 3-kinase activity	0.002
voltage-gated calcium channel activity	0.007	phosphatidylinositol 3-kinase activity	0.003
high voltage-gated calcium channel activity	0.007	1-phosphatidylinositol-3-kinase activity	0.014
protein tyrosine kinase activity	0.008	C3HC4-type RING finger domain binding	0.015
**Biological process**			
regulation of cation channel activity	8.71E-05	regulation of transport	0.004
regulation of NMDA receptor activity	0.001	photoreceptor cell development	0.005
positive regulation of behavior	0.002	eye photoreceptor cell development	0.006
regulation of skeletal muscle cell differentiation	0.002	lymphocyte differentiation	0.010
positive regulation of circadian rhythm	0.003	anion transport	0.010
**Cellular component**			
PRC1 complex	4.04E-04	unconventional myosin complex	0.026
apical dendrite	0.025	proton-transporting V-type ATPase complex	0.048
transcription factor TFIID complex	0.026		
nuclear ubiquitin ligase complex	0.043		
RNA polymerase II transcription factor complex	0.046		
**KEGG pathway**			
PPAR signaling pathway	0.018	Carbohydrate digestion and absorption	0.003
Hypertrophic cardiomyopathy (HCM)	0.019	Regulation of actin cytoskeleton	0.005
Fructose and mannose metabolism	0.024	Pyrimidine metabolism	0.011
Arrhythmogenic right ventricular cardiomyopathy (ARVC)	0.027	Focal adhesion	0.013
Glycosaminoglycan degradation	0.028	Glyoxylate and dicarboxylate metabolism	0.017

**Table 2:** Top five most significantly (*p* < 0.05) overrepresented gene ontology (GO) terms and Kyoto encyclopedia of genes and genomes (KEGG) pathways represented by either hypo- or hypermethylated differentially methylated region (DMR)-associated genes implicated by Stress treatment compared to control (*p* < 0.01).

Hypomethylated	p-value	Hypermethylated	p-value
Term		Term	
**Molecular function**			
SUMO-specific protease activity	0.005	voltage-gated potassium channel activity in ventricular cardiac muscle cell action potential repolarization	4.96E-04
protein-arginine omega-N asymmetric methyltransferase activity	0.007	voltage-gated potassium channel activity involved in cardiac muscle cell action potential repolarization	0.002
nonmembrane spanning protein tyrosine kinase activity	0.009	oxidoreductase activity, acting on NAD(P)H, quinone or similar compound as acceptor	0.004
histone-arginine N-methyltransferase activity	0.010	cytokine activity	0.006
arginine N-methyltransferase activity	0.014	peptidyl-proline 4-dioxygenase activity	0.006
**Biological process**			
histone methylation	0.003	positive regulation of protein secretion	3.88E-04
smooth muscle contraction	0.005	regulation of macrophage cytokine production	0.001
apoptotic nuclear changes	0.007	ventricular cardiac muscle cell membrane repolarization	0.001
regulation of microtubule cytoskeleton organization	0.007	positive regulation of cytokine secretion	0.001
neuron projection extension in neuron projection guidance	0.007	cardiac muscle cell membrane repolarization	0.001
**Cellular component**			
multivesicular body membrane	0.005	multivesicular body	0.025
T cell receptor complex	0.009	T cell receptor complex	0.045
actin cytoskeleton	0.017	platelet alpha granule	0.045
multivesicular body	0.021		
cytoplasmic stress granule	0.024		
**KEGG pathway**			
Chemokine signaling pathway	0.020	Cytokine-cytokine receptor interaction	1.34E-06
		Neuroactive ligand-receptor interaction	0.008
		Antigen processing and presentation	0.013
		TGF-beta signaling pathway	0.013
		Hematopoietic cell lineage	0.015

**Table 3:** Top five most significantly (*p* < 0.05) overrepresented gene ontology (GO) terms and Kyoto encyclopedia of genes and genomes (KEGG) pathways represented by either hypo- or hypermethylated differentially methylated region (DMR)-associated genes implicated by combined Ethanol + Stress treatments compared to control (*p* < 0.01).

Hypomethylated	p-value	Hypermethylated	p-value
Term		Term	
**Molecular function**			
acetylglucosaminyltransferase activity	0.001	anion transmembrane transporter activity	0.005
Rho guanyl-nucleotide exchange factor activity	0.014	inorganic anion transmembrane transporter activity	0.008
ether hydrolase activity	0.014	organic anion transmembrane transporter activity	0.009
phosphofructokinase activity	0.014	cytokine receptor activity	0.011
transmembrane receptor protein tyrosine kinase activity	0.015	extracellular ATP-gated cation channel activity	0.012
**Biological process**			
mRNA polyadenylation	4.50E-04	positive regulation of calcium-mediated signaling	0.006
RNA polyadenylation	0.001	monocarboxylic acid metabolic process	0.006
regulation of neuron migration	0.001	sphingosine-1-phosphate signaling pathway	0.009
positive regulation of neuron migration	0.002	amylin receptor signaling pathway	0.009
neuron projection extension	0.005	response to insulin	0.009
**Cellular component**			
L-type voltage-gated calcium channel complex	0.011	integral component of nuclear inner membrane	0.003
azurophil granule membrane	0.013	extrinsic component of external side of plasma membrane	0.012
		G-protein coupled receptor dimeric complex	0.020
		brush border membrane	0.035
**KEGG pathway**			
		Cytokine-cytokine receptor interaction	1.68E-05
		Hematopoietic cell lineage	0.001
		Mucin type O-glycan biosynthesis	0.001
		JAK-STAT signaling pathway	0.003
		Dilated cardiomyopathy (DCM)	0.026

### DNA Methylation Patterns Are Shared by Different Treatments

**Figure 2:**
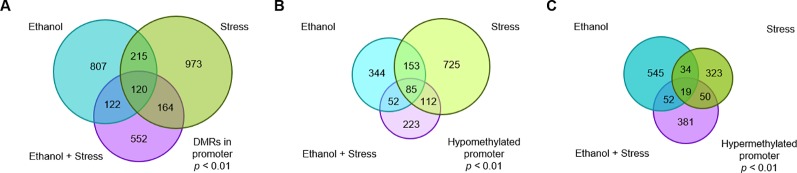
The number of genes with significant promoter DNA methylation changes shared following prenatal ethanol treatment, early life stress, or the combination of ethanol + stress with **(A)** all genes grouped together, **(B)** genes with hypomethylated promoters grouped together, and **(C)** genes with hypermethylated promoters grouped together.

While genes implicated by promoter DMRs are unique to each treatment, there are regions of overlap between treatments (**Figure 2**). 120 genes are implicated by promoter DMRs following ethanol, stress, as well as ethanol + stress (**Figure 2A**). When directionality is considered, there are 85 genes implicated by hypomethylated promoter DMRs in all three treatment groups (**Figure 2B**). Finally, there are 19 genes implicated by hypermethylated promoter DMRs in all three treatment groups (**Figure 2C**). Genes consistently implicated by promoter DMR hypomethylation tend to be important for histone methylation (*p* < 0.04) and cyclic adenosine monophosphate (cAMP) protein kinase activity (*p* < 0.04) (**Table 4**). Conversely, genes implicated by promoter DMR hypermethylation are important for ion channel activity (*p* < 0.007) as well as neuroactive ligand-receptor interactions (*p* = 0.04) and chemokine signaling (*p* < 0.003).

**Table 4:** Top five most significantly (*p* < 0.05) overrepresented gene ontology (GO) terms and Kyoto encyclopedia of genes and genomes (KEGG) pathways represented by either hypo- or hypermethylated differentially methylated region (DMR)-associated genes implicated by all three treatment categories—Ethanol, Stress, and combined Ethanol + Stress compared to control (*p* < 0.01).

Hypomethylated	p-value	Hypermethylated	p-value
Term		Term	
**Molecular function**			
cAMP-dependent protein kinase regulator activity	0.029	ATP-gated ion channel activity	0.007
protein-arginine omega-N asymmetric methyltransferase activity	0.034	extracellular ATP-gated cation channel activity	0.007
histone-arginine N-methyltransferase activity	0.038	nucleotide receptor activity	0.007
cAMP-dependent protein kinase inhibitor activity	0.038	purinergic receptor activity	0.008
arginine N-methyltransferase activity	0.042	mannose binding	0.010
**Biological process**			
endosome to lysosome transport *via* multivesicular body sorting pathway	0.025	mesodermal cell fate commitment	0.006
phagosome-lysosome fusion	0.025	bone resorption	0.008
choline catabolic process	0.025	neuromuscular process controlling balance	0.013
regulation of protein metabolic process	0.027	neuromuscular process	0.015
regulation of high voltage-gated calcium channel activity	0.029	response to interferon-alpha	0.016
**Cellular component**			
L-type voltage-gated calcium channel complex	0.029	integral component of nuclear inner membrane	0.011
perinuclear endoplasmic reticulum	0.034	multivesicular body	0.028
**KEGG pathway**			
		Chemokine signaling pathway	0.001
		Cytokine-cytokine receptor interaction	0.003
		Neuroactive ligand-receptor interaction	0.042

### DNA Methylation Alterations in Relation to Gene Expression

Since hippocampal transcriptome profiles exist for these mice, genes implicated by altered promoter methylation could be compared to genes with altered transcription. The number of genes represented by altered promoter methylation following each treatment is represented by the direction of transcriptional change following the RNA-Seq study previously reported are presented here (**Table 5**). While many genes are included for comparison, the overlap between genes implicated by altered DNA methylation and transcription is minimal. For ethanol mice, 1,264 genes with promoter DMRs were compared to the previously reported 164 unique transcripts altered in these samples. There are two corresponding transcripts with hypomethylated promoters and increased expression, *Slc2a9*, solute carrier family 2 (facilitated glucose transporter), member 9, and *Blvrb*, biliverdin reductase B (flavin reductase (NADPH)). Conversely, for hypermethylated promoters, there are two transcripts with decreased expression following PAE, *Eif4g1*, eukaryotic translation initiation factor 4, gamma 1, and *Myo6*, myosin VI.

**Table 5:** Relevant genes through the complementary direction, increase (↑) or decrease (↓) of methylation and gene expression changes compared to control for genes differentially expressed with promoter differentially methylated regions (DMRs).

	Direction				Methylation				Expression		
Group	Methylation	Gene expression	Number of genes	Gene name	DMR locus	fold change	p–value		beta	p–value	
				*Slc2a9*	chr5:38481041-38481660	−1.80	0.006		2.576	0.005	
		↑	2								
	↓			*Blvrb*	chr5:38481041-38481660	−1.80	0.006		2.576	0.005	
		↓	0	–	–	–	–		–	–	
		–	632	–	–	–	–		–	–	
Ethanol											
				*Eif4g1*	chr16:20671221-20671520	inf	0.003		−3.487	0.008	
		↓	2								
	↑			*Myo6*	chr9:80164241-80164600	3.03	0.009		−3.625	0.001	
		↑	0	–	–	–	–		–	–	
		–	648	–	–	–	–		–	–	
				Arpp21	chr9:112234421-112234980	−3.79	0.002		2.253	0.006	
		↑	3	*Mark2*	chr19:7340121-7341000	−2.03	0.001		3.413	0.003	
	↓			Rab33b	chr3:51485781-51486080	-inf	0.003		2.830	0.009	
Stress		↓	2	–	–	–	–		–	–	
		–	1070	–	–	–	–		–	–	
		↓	1	*Slc39a11*	chr11:113567341-113567720	3.33	0.010		−2.768	0.009	
	↑	↑	0	–	–	–	–		–	–	
		–	425	–	–	–	–		–	–	
				*Arpp21*	chr9:112184321-112185360	−1.05	0.006		2.663	0.001	
		↑	3	*Dlg3*	chrX:100773301-100773560	-inf	0.009		3.123	0.001	
	↓			*Pinx1*	chr14:63859301-63859580	-inf	0.007		0.439	0.005	
		↓	2	–	–	–	–		–	–	
Ethanol + Stress		–	467	–	–	–	–		–	–	
				*Slu7*	chr11:43434381-43434620	inf	0.004		−0.459	0.007	
		↓	2								
	↑			*Unc119*	chr11:78341401-78341900	1.90	0.006		−3.900	0.004	
		↑	1	–	–	–	–		–	–	
		–	499	–	–	–	–		–	–	

– Indicates data is not applicable or not provided for clarity.

In the stress group, 1,472 genes with promoter DMRs were compared to the 116 unique transcripts altered in these samples. There are three genes with hypomethylated promoters and increased transcript expression, *Arpp21*, cyclic AMP-regulated phosphoprotein, 21, *Mark2*, microtubule affinity regulating kinase 2, and *Rab33b*, RAB33B, member RAS oncogene family. One gene, *Slc39a11*, solute carrier family 39 (metal ion transporter), member 11, is hypermethylated at its promoter and has reduced transcript expression following maternal separation.

For ethanol + stress mice, 958 genes with promoter DMRs were compared to the 217 unique transcripts altered in these samples. Interestingly, *Arpp21* is also hypomethylated at its promoter and overexpressed following ethanol + stress treatment alongside two additional genes, *Dlg3*, discs large MAGUK scaffold protein 3, and *Pinx1*, PIN2/TERF1 interacting, telomerase inhibitor 1. Conversely, two genes share increased promoter methylation and decreased gene expression, *Slu7*, pre-mRNA-splicing factor SLU7, and *Unc119*, unc-119 lipid binding chaperone. While the number of genes with altered promoter methylation and expression resulting from each treatment or combination of treatments is minimal, most of these genes have known brain functions in the literature, ranging from neuronal migration to synapse formation and plasticity (**Table 6**).

**Table 6:** Genes of interest identified by altered promoter methylation and gene expression with literature-based evidence of central nervous system function.

Gene of interest	Central nervous system relevance
*Slc2a9*	Memory performance (Houlihan et al., 2010) Social phobia (Lyngdoh et al., 2013)
*Eif4g1*	Neurodegeneration (Dhungel et al., 2015)
*Myo6*	Dendritic spine length (Avraham et al., 1995) Postsynaptic receptor internalization (Osterweil et al., 2005) Presynaptic glutamate release potentiation (Yano et al., 2006)
*Arpp21*	Dendrite growth (Rehfeld et al., 2018)
*Mark2*	Neuron migration (Sapir et al., 2008; Mejia-Gervacio et al., 2012)
*Rab33b*	Axon growth (Huang et al., 2019)
*Dlg3*	Synapse formation, spatial learning (Cuthbert et al., 2007) Intellectual disability (Tarpey et al., 2004)
*Pinx1*	Late-onset Alzheimer disease (Tosto et al., 2019)
*Unc119*	Dendritic branching (May et al., 2014)

## Discussion

FASD is a prevalent societal burden that has remained complex to understand. Children born with FASD are often exposed to stressful postnatal environments that include maternal separation. Our main objective was to understand how early life stresses may complicate molecular changes underlying behavioral deficits that arise following prenatal ethanol exposure. Previously, behavioral changes resulting from prenatal ethanol exposure and postnatal maternal separation stress have been characterized (Alberry and Singh, 2016), with lasting changes to the hippocampal transcriptome also being reported (Alberry et al., 2019). Here we focus on alterations to promoter DNA methylation in the same mice. The behavioral deficits originally reported were subtle in nature, a direct result of the subtlety of the treatment paradigms used. In addition, gene expression alterations were small in fold change, detected at postnatal day 70, long after the final experimental interventions.

### Ethanol-Induced Alterations in DNA Methylation

Prenatal ethanol exposure results in specific changes in promoter DNA methylation. We report promoter hypomethylation of genes important for brain function and formation as well as for transcriptional regulation following prenatal ethanol exposure. We also found promoter hypermethylation of genes important for phosphoinositide kinase activity as well as cytoskeleton regulation. These alterations may be lasting evidence of altered control of gene expression earlier during development that persists into adulthood. The ethanol exposure model employed here has been used previously, with lasting alterations to whole-brain DNA methylation profiles (Laufer et al., 2013). While we report comparable amounts of hypo- and hypermethylated gene promoters following continuous gestational ethanol exposure, previous work using targeted binge exposure trimester three equivalent models have found alcohol-induced hypermethylation in the hippocampus in both mice (Chater-Diehl et al., 2016) and rats (Otero et al., 2012). Conversely, a model of ethanol exposure more representative of trimester two equivalent exposure in mice found global hypomethylation in the neocortex, but this pattern is more complex when brain structures are independently assessed (Öztürk et al., 2017). Our work uses a continuous exposure model and is aimed at investigating promoter methylation, highlighting the importance of dose and timing of ethanol exposure as well as specific methylation patterns being observed.

### Maternal Separation-Induced Alterations in DNA Methylation

We report more genes with hypo- than hypermethylated promoters following early life stress *via* maternal separation. Of these, hypomethylated genes tend to be important for histone methylation and chemokine activity. Meanwhile, hypermethylated promoters tend to be of genes important for cytokine interactions. Early life stress results in lasting changes to DNA methylation in the hippocampus of rats (Kosten et al., 2014). In humans, child abuse has been associated with hypermethylation of several genomic loci in the hippocampus (McGowan et al., 2009). Meanwhile, maternal separation in rats induces an inflammatory immune response in the hippocampus (Roque et al., 2016). Interestingly, early life stress has been associated with reduced serum cytokine levels, suggesting that hypermethylation of related genes may not be hippocampal specific, rather playing a more ubiquitous role in the body (Raineki et al., 2017).

### Ethanol + Maternal Separation-Induced Alterations in DNA Methylation

The combination of prenatal ethanol exposure and maternal separation stress resulted in the hypomethylation of genes important for neuronal migration and hypermethylation of genes related to cytokine interactions. Similarly, genes that were implicated by hypermethylated promoters following any of the treatments were also important for chemokine and cytokine interactions. It is apparent from **Figure 1** that the addition of early life stress to individuals that faced prenatal ethanol exposure does not result in an additive DNA methylation response. Few genes are shared between ethanol only or stress only and the combination of treatments as implicated by promoter DMRs. Prenatal ethanol exposure followed by early life stress has been associated with altered immune system development, with early life adversity differentially affecting peripheral and central immune response depending on previous ethanol exposure (Raineki et al., 2017).

### Overlaps Between DNA Methylation and Gene Expression Changes Are Scarce

We previously reported changes in gene expression in association with behavioral deficits following prenatal ethanol exposure and maternal separation in these mice (Alberry et al., 2019). Interestingly the module of genes most closely associated with treatment and anxiety-like behavior includes genes important for transcriptional regulation and neurodevelopment. With these results in mind, we examined what overlap exists between genes with altered expression and altered promoter methylation that is complementary.

Following prenatal ethanol exposure, two genes have decreased promoter methylation and corresponding increased expression—*Slc2a9* and *Blvrb*. *Slc2a9* codes for a fructose transporter that acts as a uric acid transporter (Le et al., 2008). While not much is known regarding its role in the brain, SLC2A9 SNPs have been associated with better memory performance (Houlihan et al., 2010), and social phobia (Lyngdoh et al., 2013). *Blvrb* codes for an oxidoreductase important for heme degradation, with no clear link to brain function. Prenatal ethanol exposure results in promoter hypermethylation and decreased transcript expression for two genes, *Eif4g1* and *Myo6*. *Eif4g1* produces a translation initiation factor, eIF4G, with mutations present in familial Parkinson Disease (Chartier-Harlin et al., 2011). In fact, this gene acts as a scaffold protein with others in the hippocampus in ways that are perturbed in neurodegeneration likely through protein misfolding (Dhungel et al., 2015). *Myo6* is an actin-based motor protein that has been previously linked to stress response in the mouse hippocampus (Tamaki et al., 2008). Mice lacking this gene have fewer synapses and shorter dendritic spines (Avraham et al., 1995). *Myo6* is important for postsynaptic stimulus-dependent α-amino-3-hydroxy-5-methyl-4-isoxazole propionic acid-type glutamate receptor (AMPAR) internalization (Osterweil et al., 2005), as well as for brain-derived neurotrophic factor (BDNF)-mediated glutamate release from presynaptic terminals in long-term potentiation (Yano et al., 2006). Together, these results suggest the combined accumulation of DNA methylation, followed by reduced expression of these transcripts may be important for the altered brain-related phenotypes observed in these mice following prenatal ethanol exposure.

There are three genes with promoter hypomethylation and a corresponding increase in transcript expression following maternal separation stress, *Arpp21*, *Mark2*, and *Rab33b*. *Arpp21* is also implicated by the combination of ethanol + stress through promoter hypomethylation and increased expression. The ARPP21 protein product is composed of conserved domains for RNA-binding, with the ability to interact with eIF4G and preferentially bind to the 3' untranslated regions of mRNAs. Additionally, overexpression and knockdown experiments of ARPP21 show it positively regulates dendrite growth (Rehfeld et al., 2018). While we report increased *Arpp21* expression following stress or ethanol + stress, stress-induced decreases have also been reported (Constantinof et al., 2019). *Mark2* produces a serine/threonine-protein kinase involved in cell polarity and microtubule dynamics, as well as in neuron migration (Sapir et al., 2008; Mejia-Gervacio et al., 2012). *Rab33b* is a member of Rab family proteins that regulate intracellular vesicular trafficking, particularly at the Golgi apparatus (Zheng et al., 1998). Recently, the zebrafish ortholog of *Rab33b*, *rab33ba*, has been shown to mediate axon outgrowth in the forebrain (Huang et al., 2019). *Slc39a11* is the only gene that has promoter hypermethylation and overexpression in the stress group. It codes for a Zrt-, Irt-like protein (ZIP) for zinc transport (Yu et al., 2013). Taken together, these results suggest that the loss of methylation and subsequent overexpression of these genes following postnatal maternal separation stress may lead to observed changes in behavioral phenotypes mediated by improper neuronal migration and growth.

The combination of ethanol + stress treatments results in three genes with increased expression and corresponding promoter hypomethylation, *Arpp21*, *Dlg3*, and *Pinx1*. *Arpp21* was introduced previously as it was also implicated by stress alone. Of these, *Dlg3* is one of the most significantly altered transcripts in response to Stress and Ethanol + Stress treatments that we previously reported (Alberry et al., 2019). It codes for synapse-associated protein 102, a protein important for synapse formation. Knockout mice have impaired synaptic plasticity and spatial learning (Cuthbert et al., 2007), while human *DLG3* mutations result in nonsyndromic X-linked intellectual disability (Tarpey et al., 2004). *Pinx1* is a telomerase inhibitor with little known brain function, although variants have been associated with late-onset Alzheimer disease (Tosto et al., 2019). Two genes share decreased promoter methylation and increased expression following the combination of treatments, *Slu7* and *Unc119*. *Slu7* is a pre-mRNA splicing factor that forms part of the spliceosome and is important for genome stability (Jiménez et al., 2019). *Unc119* codes for a lipid binding chaperone required for G protein localization in sensory neurons (Zhang et al., 2011). In fact, knockdown of *Unc119* results in neurotoxicity and decreased dendritic branching (May et al., 2014). These results suggest that dysregulation of genes important for neuronal migration and synapse formation as well as genome integrity *via* altered promoter methylation following the combination of ethanol + stress may underlie the complex and heterogeneous behavioral changes observed in these mice.

### Beyond Promoter DNA Methylation

Promoter DNA methylation is an essential mechanism of epigenetic control for the expression of genes. It is affected by environmental factors, including ethanol and stress. Changes in promoter DNA methylation status can lead to the initiation and maintenance of gene expression over time. Promoter DNA methylation has been the research focus in FASD, however, DNA methylation does not act in isolation. While we focus on DMRs in gene promoters, methylation status at a single CpG site may be sufficient to influence gene expression. In this analysis, we may miss the influence of a single CpG that has this type of influence without resulting in a significantly altered region of DNA methylation. Similarly, we have focused on promoter DNA methylation for its known influence on gene expression *via* altered transcription factor binding. Gene body or other intergenic changes in methylation would not be captured in this analysis and may be important for gene expression. Phenotypic outcomes are often the result of interrelated epigenetic mechanisms. For example, noncoding RNAs are also important for the regulation of gene expression at the transcriptional and posttranscriptional levels. They include long noncoding RNAs (lncRNAs) (Lee, 2012), microRNAs (miRNAs) (Chuang and Jones, 2007), short interfering RNAs (siRNAs), and piwi-interacting RNAs (piRNAs) (Holoch and Moazed, 2015). In FASD research, the noncoding RNA research has been focused on the relationship between microRNAs and their mRNA gene targets. Several microRNAs are altered in reciprocal direction to their gene targets following prenatal alcohol exposure (Balaraman et al., 2013; Laufer et al., 2013), offering another potential avenue for control. Additionally, modifications to histone tails at specific genomic loci are the foundation of epigenetic mechanisms, with a wide variety of known potential modifications (Allis and Jenuwein, 2016). In FASD mouse models, altered histone modifications (Veazey et al., 2015; Chater-Diehl et al., 2016), as well as enhanced lysine dimethyltransferase activity resulting in increased histone H3 lysine 9 and 27 dimethylation and neurodegeneration have been reported (Subbanna et al., 2013). Such results suggest changes in gene-specific expression in response to prenatal ethanol exposure must be the result of a variety of epigenetic mechanisms. Although DNA methylation, noncoding RNAs, and histone modifications have all been implicated, their inter-relationship is not known.

The promoter DNA methylation results included in this report are novel in that they were obtained from the same mice that were used to assess behavioral changes (Alberry and Singh, 2016) and hippocampal gene expression (Alberry et al., 2019). This allows for assessment of the relationship between promoter DNA methylation and expression of that gene in response to prenatal ethanol exposure, postnatal maternal separation, and the combination of both treatments. Our results implicate many genes *via* altered expression or promoter DNA methylation, and these genes are relevant in the context of their involvement in ethanol or stress response. In fact, many genes implicated are important for brain development and function. What is apparent is that changes in expression of only a small subset of genes is compatible with changes in promoter DNA methylation. The results argue that promoter methylation is only partly involved in the environmentally induced changes in gene expression we have modeled. Epigenetic mechanisms other than promoter DNA methylation therefore are likely responsible for the lasting gene expression alterations and behavioral abnormalities seen in pups subjected to prenatal ethanol exposure, maternal separation, and the combination of the two treatments. Although logical, such conclusions must be assessed experimentally, as the hippocampus represents a heterogenous cell population. Also, the interventions used are relatively mild, applied during neurodevelopment, with methylation status assessed in adulthood. Not surprisingly, this experimental paradigm has resulted in subtle changes in both gene expression and DNA methylation. Future research must focus on experimentally establishing mechanisms involved in altered gene expression and maintenance of these changes in different cell types within the brain. This will require modified experimental protocols and the use of single cell sequencing. Greater understanding into how these changes occur and persist has the potential to lead to the development of novel strategies for the amelioration of FASD-related deficits for children born with this common and complex disorder.

## Data Availability Statement

The datasets generated for this study can be found at the Gene Expression Omnibus (GEO), accession numbers GSE137984 and GSE133369 for MeDIP-Seq and RNA-Seq experiments, respectively.

## Ethics Statement

The animal study was reviewed and approved by Animal Use Subcommittee at Western University.

## Author Contributions

BA and SS have made substantial contributions to the conception and design of the work. BA was responsible for data acquisition and analysis. All authors completed data interpretation. BA created the first manuscript draft. All authors substantively revised it and have approved the submitted version.

## Funding

This work has been supported by a Discovery Research Grant from the Natural Sciences and Engineering Research Council of Canada awarded to SS.

## Conflict of Interest

The authors declare that the research was conducted in the absence of any commercial or financial relationships that could be construed as a potential conflict of interest.

## Supplementary Material

Supplementary Table 1 Hypo- and hypermethylated promoter DMRs from adult hippocampus following prenatal ethanol exposure compared to untreated controls filtered by significance (*p* < 0.01).

Supplementary Table 2 Hypo- and hypermethylated promoter DMRs from adult hippocampus following postnatal maternal separation stress treatment compared to controls filtered by significance (*p* < 0.01).

Supplementary Table 3 Hypo- and hypermethylated promoter DMRs from adult hippocampus following prenatal alcohol exposure and postnatal maternal separation stress as compared to controls, filtered by significance (*p* < 0.01).
